# Robotic-Assisted Ascending Hemicolectomy for Colon Cancer in a Patient With Situs Inversus Totalis: A Case Report

**DOI:** 10.7759/cureus.92098

**Published:** 2025-09-11

**Authors:** Martin Infante Altamirano, Marianna Zeichen, Rohan G Kulangara, Henry J Lujan

**Affiliations:** 1 General Surgery, Universidad Peruana de Ciencias Aplicadas, Lima, PER; 2 Colorectal Surgery, Jackson Memorial Hospital, Miami, USA; 3 General Surgery, Southwest Surgical Associates, Houston, USA

**Keywords:** ascending colon, cancer colon, hemi-colectomy, robotic colorectal surgery, situs inversus totalis (sit)

## Abstract

We present a case of robotic-assisted ascending hemicolectomy in an 82-year-old woman with situs inversus totalis. The patient underwent a screening colonoscopy, during which a polypoid lesion in the ascending colon was discovered. Biopsy results revealed a tubular adenoma with high-grade dysplasia. An ascending hemicolectomy with radical lymphadenectomy was subsequently performed, leading to a final diagnosis of a tubulovillous adenoma with focal intramucosal carcinoma, negative for metastatic disease. The challenges related to instrument handling during surgery in patients with situs inversus totalis can be mitigated by using a robotic approach, which preserves the efficiency of laparoscopic techniques. This report highlights the importance of robotic surgery in managing complex procedures in patients with anatomical variations or other technical challenges.

## Introduction

Situs inversus totalis (SIT) is a rare congenital anomaly characterized by a complete mirror-image transposition of the thoracic and abdominal viscera [1]. Its low incidence and the associated anatomical variations can increase the complexity of surgical procedures. Laparoscopic surgery has been reported in such cases, but it presents challenges involving instrument handling. The potential advantages of robotic surgery in this patient population have not been extensively documented. To our knowledge, this is only the second reported case of a robotic ascending hemicolectomy performed in a patient with SIT. The purpose of this report is to highlight possible intraoperative adjustments that may enhance surgical efficiency to optimize such cases from a technical standpoint. 

## Case presentation

An 82-year-old woman underwent a screening colonoscopy, which revealed a 40 mm polypoid lesion in the ascending colon. Biopsies demonstrated a tubular adenoma with high-grade dysplasia. Her past medical history was notable for SIT, and her body mass index (BMI) was 28 kg/m^2^. Based on these findings, a robot-assisted ascending hemicolectomy with radical lymphadenectomy was performed.

**Figure 1:**
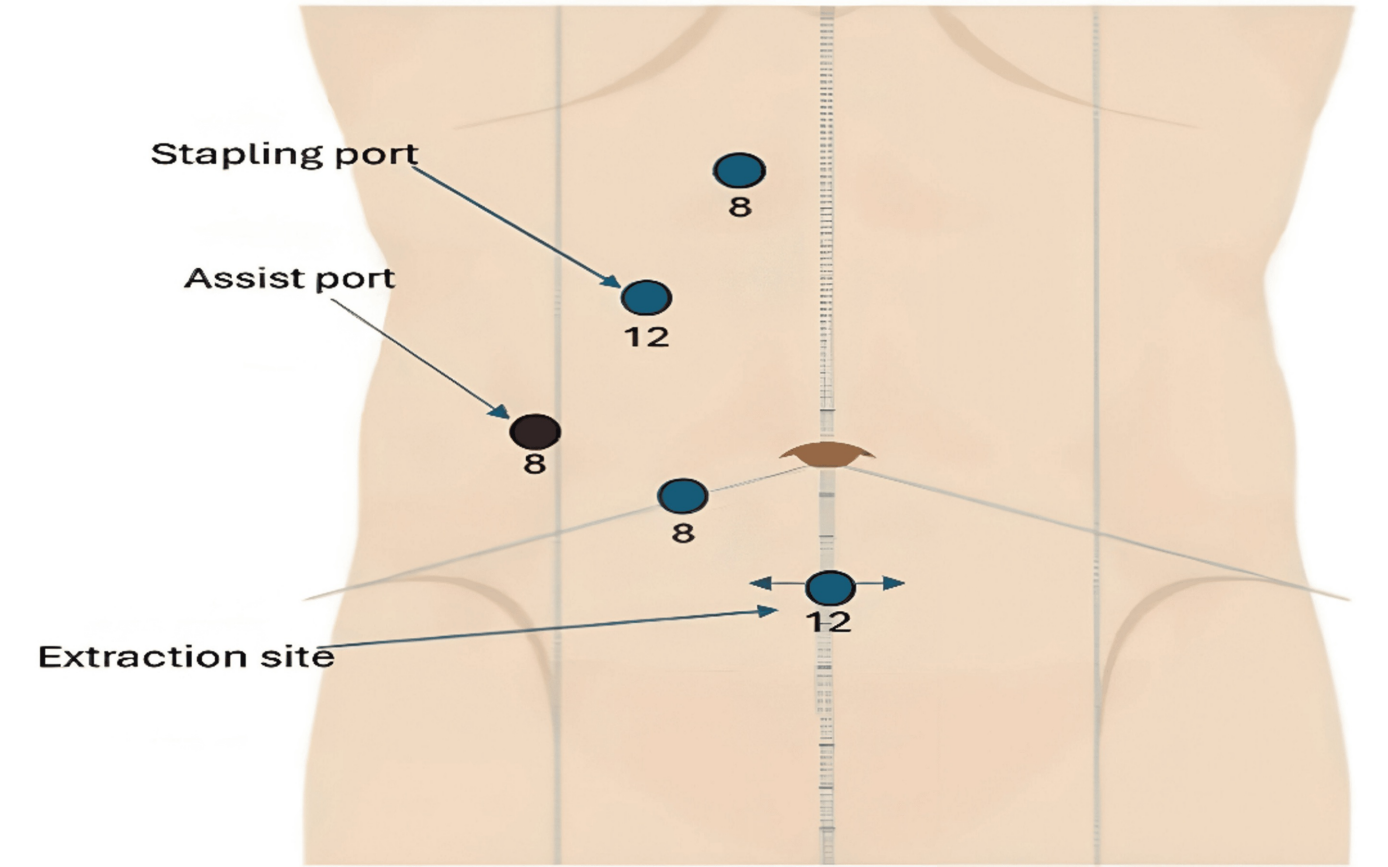
Position of trocars and arm configuration settings for the Da Vinci® Xi system (Intuitive Surgical, Inc.). Image Credit: Authors

The patient was positioned supine, and a mirror-image port configuration was utilized. Trocars were placed in a linear arrangement from the suprapubic region towards the right shoulder, with a slight curvature due to anatomical constraints. Initially, a 12 mm suprapubic trocar was inserted. Two 8 mm trocars were placed paraumbilical and in the right upper abdomen, followed by a 12 mm trocar adjacent to the latter. A 5 mm assistant AirSeal® port (ConMed Corporation, Utica, New York, United States) was positioned equidistant between the two middle ports (Figure 1).

**Figure 2:**
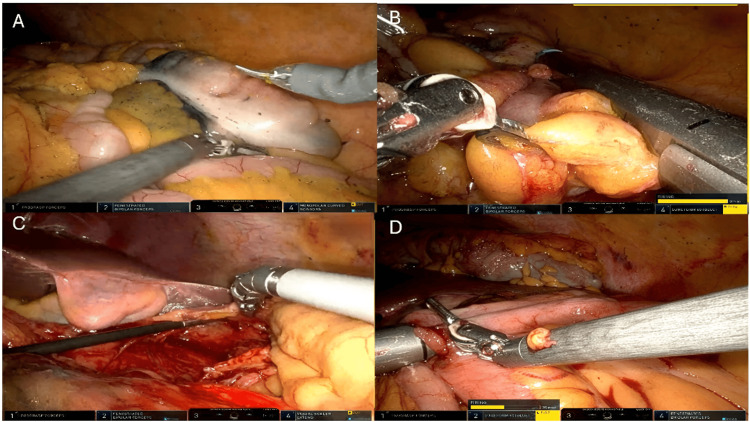
Intraoperative steps of robotic-assisted hemicolectomy A. Visualization of the tattoo mark in the ascending colon; B. Robotic stapler-assisted transection of the terminal ileum; C. Mobilization of the hepatic flexure with the gallbladder visible in the left upper quadrant; D. Intracorporeal anastomosis performed with a robotic stapler

The Da Vinci Xi Surgical System (Intuitive Surgical, Inc., Sunnyvale, California, United States) was employed. The patient was placed in reverse Trendelenburg position with the left side elevated, and the robot was docked from the patient's left. A 30° endoscope (third arm) was used, with ProGrasp™ forceps (first arm) (Intuitive Surgical, Inc.) for mobilization, fenestrated bipolar forceps (second arm), and a vessel sealer (fourth arm). Dissection commenced in a medial to lateral fashion, starting with the ileocolic pedicle. Division of the ileocolic vessels and transection of the terminal ileum and transverse colon were performed using a robotic stapler via the fourth arm. A tension-free, safe, intracorporeal stapled anastomosis was performed with the stapler introduced through the second arm (Figure 2).

The specimen was extracted through a small suprapubic incision covered by a wound protector. The estimated blood loss was 5 ml. The patient was discharged without complications on postoperative day three, after passing flatus and tolerating a low-residue diet. No readmission occurred. Final histopathology revealed a 3.5 cm tubulovillous adenoma with focal intramucosal carcinoma. All 18 retrieved lymph nodes were negative for metastatic disease, consistent with pTisN0 disease. 

## Discussion

SIT is a rare congenital anomaly, with an estimated incidence of approximately one in 10,000 individuals, and a higher prevalence in males [1,2]. Although visceral transposition occurs, the peripheral nervous system remains unaltered, which can present unique challenges in both diagnostic evaluation and surgical management [3]. In the present case, the patient was undergoing a screening colonoscopy when a polypoid lesion in the ascending colon was biopsied. The diagnosis of SIT was already established, and preoperative review of prior abdominal imaging was undertaken to facilitate surgical planning. Detailed evaluation of the anatomical configuration is essential in such cases to anticipate potential intraoperative difficulties. Three-dimensional (3D) computed tomography (CT) and CT angiography have been described as valuable tools for accurately delineating the visceral and vascular anatomy, thereby aiding in the optimization of the operative strategy [4].

To date, five case reports have described the use of robotic surgery for colorectal cancer in patients with SIT, only one of which involved colon cancer. In that report, port placement was arranged as a mirror image of the standard configuration for a right hemicolectomy, utilizing four 8 mm and two 12 mm trocars [4]. In the present case, we employed a similar mirror-image alignment, with a slight curvature necessitated by the patient’s specific anatomy. Although some reports note technical challenges for right-handed surgeons, particularly when manipulating energy devices with the left hand or operating pedals with the left foot in SIT patients, the robotic platform helped mitigate these limitations, enabling flexible and safe instrument handling [5]. 

Intracorporeal anastomosis during right hemicolectomy permits the use of a smaller extraction site, which can be placed in the lower abdomen, thereby reducing postoperative pain, surgical site infection rates, and the risk of incisional hernia [6,7]. Meticulous tissue handling, combined with the creation of a tension-free and well-perfused anastomosis, is critical in minimizing the risk of anastomotic leakage [8]. In the present case, an intracorporeal anastomosis was initially attempted using the robotic stapler through the suprapubic 12 mm trocar. However, despite this port being utilized for both bowel and vessel transection, the stapler’s angulation proved suboptimal. Consequently, the decision was made to introduce the stapler through the right upper quadrant 12 mm port to facilitate a safer and more ergonomic anastomosis.

This case demonstrates how robotic surgery facilitates the technical execution of complex procedures in patients with SIT by compensating for ergonomic challenges and enabling tailored port placement. Preoperative anatomical evaluation and intraoperative adaptability remain crucial to ensuring surgical safety and optimizing outcomes in this unique patient population.

## Conclusions

We successfully performed a safe and efficient robotic right hemicolectomy in a patient with situs inversus totalis. The robotic platform facilitates adaptation of surgical techniques to accommodate anatomic variations while maintaining procedural proficiency and achieving comparable oncological and clinical outcomes. A mirror-image port configuration, with minor adjustments tailored to the patient’s anatomy, proved adequate for performing this type of procedure.
